# The burden of headache disorders in the adult population of Mongolia: estimates, and a health-care needs assessment, from a cross-sectional population-based study

**DOI:** 10.1186/s10194-024-01856-6

**Published:** 2024-09-09

**Authors:** Otgonbayar Luvsannorov, Byambasuren Tsenddorj, Dorjkhand Baldorj, Selenge Enkhtuya, Delgermaa Purev, Andreas Husøy, Timothy J. Steiner

**Affiliations:** 1 Department of Neurology, Mongolian National University of Medical Sciences, Ulaanbaatar, Mongolia; 2 Department of Neurology, State Third Central Hospital, Ulaanbaatar, Mongolia; 3 Division of Neurology, Мungunguur Clinical Hospital, Ulaanbaatar, Mongolia; 4 Department of Neuromedicine and Movement Science, Norwegian University of Science and Technology, Edvard Griegs Gate, Trondheim, Norway; 5 Department of Neurology, University of Copenhagen, Copenhagen, Denmark; 6 Division of Brain Sciences, Imperial College London, London, UK

**Keywords:** Headache disorders, Migraine, Tension-type headache, Medication-overuse headache, Epidemiology, Burden of disease, Population-based survey, Health-Care needs assessment, Mongolia, Western pacific region, Global Campaign against Headache

## Abstract

**Background:**

Having previously shown headache disorders to be prevalent in Mongolia, here we elaborate on headache as a public-health concern in this country, reporting symptom burden and headache-attributed impaired participation at individual and societal levels, and conducting a health-care needs assessment.

**Methods:**

The study followed the standardized methodology developed by the Global Campaign against Headache, generating a representative general-population sample through multi-level randomized cluster sampling. Participants aged 18–65 years were interviewed at unannounced household visits by interviewers administering the HARDSHIP questionnaire. Symptom burden was established through questions on frequency, duration and intensity of headache, with proportion of time in ictal state calculated from frequency and duration. Individual impaired participation was established through the HALT questionnaire, enquiring into lost time from paid and household work and from leisure activities. Symptom burden and impaired participation yesterday were also assessed in those reporting headache yesterday. Population-level estimates were derived by factoring in prevalence.

**Results:**

The total sample included 2,043 participants. Those reporting any headache in the last year (*n* = 1,351) spent, on average, 9.7% of all their time with headache, losing 1.3 workdays and 2.4 household days/3 months. These losses were considerably higher among those with probable medication-overuse headache (37.5%, 3.5 workdays, 6.7 household days) or other headache on ≥ 15 days/month (H15+) (21.9%, 2.4 workdays, 5.1 household days). At population-level (including those with and without headache), 6.2–7.4% of all time was spent with headache, 3.1% with H15+; 0.8 workdays and 1.4 household days/person/3 months were lost to headache, 0.3 workdays and 0.6 household days to migraine (the biggest contributor of all headache types). Our needs assessment estimated that one third (33.2%) of the adult population of Mongolia have headache (mostly migraine or H15+) likely to benefit from health care.

**Conclusion:**

This first population-based study on headache burden in Mongolia shows high levels of individual and societal burden, with H15 + the cause of greater burden at population level than migraine and TTH combined. Migraine, however, has the biggest impact on the nation’s productivity. From a purely economic perspective, Mongolia, with limited health resources, would probably be best served by focusing on mitigating migraine-attributed burden.

## Background

This manuscript continues the series of population-based studies of headache-attributed burden among adults, conducted within the Global Campaign against Headache [1–12]. It reports a study from Mongolia, a country in the Western Pacific Region (WPR). It builds upon our previously published prevalence data from this country, which showed headache disorders to be common, with a very high prevalence of disorders characterized by headache on ≥ 15 days/month (H15+) [13]. The report noted that these findings carried messages for national health policy. However, for health policy to be fully informed, both the data on associated burden and a health-care needs assessment reflecting these data were required. Our purpose here was to provide these.

This was the first such study in Mongolia, and second of its type conducted by the Global Campaign in WPR, following a study in China [9]. The findings therefore add to our knowledge and understanding of the global burden of headache.

## Methods

Full details of the study design, interview process and data collection and management have been published [13]. In summary, guided by a pilot study (to ensure feasibility), a cross-sectional population-based study was conducted during August to November 2017 among randomly selected adults (18–65 years) living in Mongolia. The study used standardized methods and questionnaire developed by the Global Campaign [14, 15]. Representativeness was achieved through multi-level randomized cluster-sampling [13]. Face-to-face interviews during unannounced home visits were performed using the Headache-Attributed Restriction, Disability, Social Handicap and Impaired Participation (HARDSHIP) questionnaire [15] translated into Mongolian language following the Global Campaign’s translation protocol [16].

### Headache diagnoses

After a neutral screening question (“have you had a headache in the last year”), participants were asked the diagnostic questions incorporated into HARDSHIP [15], based on ICHD-3 criteria [17]. Diagnoses made algorithmically included probable medication overuse headache (pMOH), other H15+, definite migraine, definite tension-type headache (TTH), probable migraine and probable TTH, made in that order. Definite and probable migraine, and definite and probable TTH, were combined for analyses. Only one diagnosis was made in each participant: those reporting more than one type of headache were asked to respond according to the type that was most bothersome [13].

### Burden measures

#### Individual burden

Symptom burden was recorded in terms of headache frequency, usual duration and usual intensity of headache. Frequency was reported in days/month, and duration in hours, both treated as continuous variables. Intensity was reported as a categorical variable, “mild”, “moderate” or “severe”, and converted to a numerical scale 1–3.

From these primary measures, two secondary measures were derived: proportion of time in ictal state (pTIS) and headache-attributed lost health. pTIS was calculated as frequency*duration (with duration capped at 24 h since frequency was reported in days/month) divided by total time (30*24 hours). Lost health was estimated, in those with migraine or TTH, as pTIS*DW, where DW was the disability weight provided by the Global Burden of Disease study [18] for the ictal state of the disorder.

Enquiry included symptom burden yesterday (duration and intensity of headache) in those reporting headache yesterday (HY) [15]. pTIS yesterday was calculated as the quotient of duration and 24.

We estimated impaired participation in paid and household work over the preceding 3 months using the Headache-Attributed Lost Time (HALT) questionnaire [19]. The methodology has been described in detail [19]. Days absent from work were added to those with less than half achieved while at work, both counted as lost days. In counterbalance, any days with more than half achieved were ignored. A similar approach was applied to household work (less than half achieved equated to nothing achieved, while more than half achieved was ignored) [19]. We also counted the number of days in which leisure activity was missed.

Impaired participation yesterday in those with HY was reported more directly, as everything, more than half, less than half or nothing achieved, making no distinction between paid work, household work and leisure time. As with HALT, less than half and nothing were summed and counterbalanced by summing more than half with everything achieved.

To assess quality of life (QoL), HARDSHIP incorporated WHOQoL-8 [20]. The unitless summed score (possible range of 8–40, with higher scores indicating better QoL) was treated as a continuous variable.

#### Population-level burden

pTIS and headache-attributed impaired participation at population level were calculated from means at individual level, factoring in headache prevalence and adjusting for age and gender. Independent estimates were made based on 1-year and 1-day prevalences. From HALT data, separate estimates were made for the three domains of participation: work, household and leisure. From HY data, such distinction was not possible (thus reflecting overall impaired participation only).

### Health-care needs assessment

We defined “need” for health care in terms of numbers likely to benefit from effective provision of health care. Accordingly, we counted everyone with H15+ (whether pMOH or other), all participants with migraine reporting headache frequency of ≥ 3 days/month, and all participants with migraine or TTH meeting at least one of the following two criteria: (a) pTIS > 3.3% (i.e., > 1 day/month) *and* usual intensity ≥ 2 (i.e., moderate or severe); (b) lost participation over the preceding 3 months from *either* paid or household work of ≥ 3 days. Double counting was avoided. Age- and gender-adjusted prevalences were calculated.

### Statistics and analyses

Continuous variables were described using means, standard errors of the mean (SEMs) and medians. In comparisons between genders and between headache types, we used ANOVA tests for continuous variables, chi-squared tests for categorical variables.

Statistical analyses were performed using SPSS version 28 (SPSS, INC, Chicago, IL). We considered *p* < 0.05 as significant.

## Results

The sociodemographic characteristics of the sample (*N* = 2,043), and headache prevalences, have already been reported [13]. The participating proportion was 98.3%. Males were somewhat under-represented in the sample (39.8%, vs. 49.2% in the national population in 2017 [21]), for which corrections were necessary. The crude 1-year prevalence of any headache was 66.1%, higher in females (73.3%) than males (55.4%). Age- and gender-adjusted prevalences were 23.1% for migraine, 29.1% for TTH, 5.7% for pMOH and 5.0% for other H15+ [13]. HY was reported by 20.1% (males 12.2%, females 25.3%) [13].

### Individual burden

#### Symptom burden

Table 1 shows the measures of symptom burden by headache type and gender. Study participants with any headache spent, on average, 9.7% of all their time with headache, but, also on average, no headache type was rated more than mild-to-moderate. Migraine was more burdensome than TTH on all measures in both genders (in particular, duration and pTIS were double), although none were tested for significance (Table 1). pMOH and other H15 + were, of course, much more frequent, with commensurately higher values of pTIS. There were no gender-related differences of interest (Table 1).

**Table 1:** Symptom burden by headache type and gender

Headache type	Overall	Male	Female	Male vs. female
	Mean±SEM, median			
**Frequency** (days/month)				
Any headache	7.0±0.2, 4.0	6.4±0.3, 3.0	7.3±0.2, 4.0	*p* = 0.03
pMOH	22.0±0.5, 20.0	21.9±0.9, 20.0	22.0±0.6, 20.0	*p* = 0.92
Other H15+	17.8±0.5, 15.0	18.2±1.2, 16.0	17.7±0.6, 15.0	*p* = 0.72
Migraine	4.5±0.2, 3.0	4.8±0.3, 3.0	4.4±0.2, 3.0	*p* = 0.38
TTH	3.6±0.1, 3.0	3.3±0.2, 2.0	3.8±0.2, 3.0	*p* = 0.03
**Duration** (hours)				
Any headache	11.2±0.5, 4.0	9.9±0.7, 3.0	11.8±0.6, 4.0	*p* = 0.06
pMOH	12.9±1.2, 7.0	14.1±2.3, 7.0	12.4±1.4, 7.0	*p* = 0.52
Other H15+	9.0±0.9, 4.0	8.2±1.7, 3.0	9.3±1.1, 4.0	*p* = 0.64
Migraine	15.7±1.0, 6.0	14.2±1.4, 6.0	16.2±1.2, 6.0	*p* = 0.37
TTH	7.4±0.5, 3.0	7.1±0.8, 3.0	7.6±0.7, 3.0	*p* = 0.61
**Intensity** (mild, moderate, severe, equated to 1, 2, 3)				
Any headache	747-513-53 (mean = 1.5)	266-149-14 (mean = 1.4)	481-364-39 (mean = 1.5)	*p* = 0.03
pMOH	38-66-17 (mean = 1.8)	14-20-4 (mean = 1.7)	24-46-13 (mean = 1.9)	*p* = 0.59
Other H15+	61-54-9 (mean = 1.6)	13-12-4 (mean = 1.7)	48-42-5 (mean = 1.6)	*p* = 0.30
Migraine	210-270-21 (mean = 1.6)	57-68-5 (mean = 1.6)	153-202-16 (mean = 1.6)	*p* = 0.87
TTH	433-122-5 (mean = 1.2)	181-48-1 (mean = 1.2)	252-74-4 (mean = 1.3)	*p* = 0.56
**Proportion of time in ictal state** (%)				
Any headache	9.7±0.5, 2.2	8.8±0.9, 1.6	10.1±0.6, 2.7	*p* = 0.22
pMOH	37.5±3.3, 22.2	40.4±6.0, 29.2	36.2±3.9, 20.8	*p* = 0.55
Other H15+	21.9±2.3, 10.4	20.9±4.8, 10.4	22.2±2.6, 11.1	*p* = 0.81
Migraine	6.9±0.4, 3.3	7.0±0.8, 3.3	6.9±0.5, 2.9	*p* = 0.96
TTH	3.4±0.3, 0.8	3.0±0.4, 0.8	3.7±0.4, 1.0	*p* = 0.27
**Headache-attributed lost health** (%)				
Migraine	3.0±0.2, 1.4	3.0±0.4, 1.4	3.0±0.2, 1.2	*p* = 0.96
TTH	0.1±0.0, 0.0	0.1±0.0, 0.0	0.1±0.0, 0.0	*p* = 0.27

pMOH: probable medication-overuse headache; H15+: headache on ≥ 15 days/month; TTH: tension-type headache

Lost health, estimated using DWs for the ictal state from GBD [18], was 3.0% for migraine in both genders and 0.1% (the lower limit of estimation) for TTH (Table 1).

### Impaired participation

**Fig. 1:**
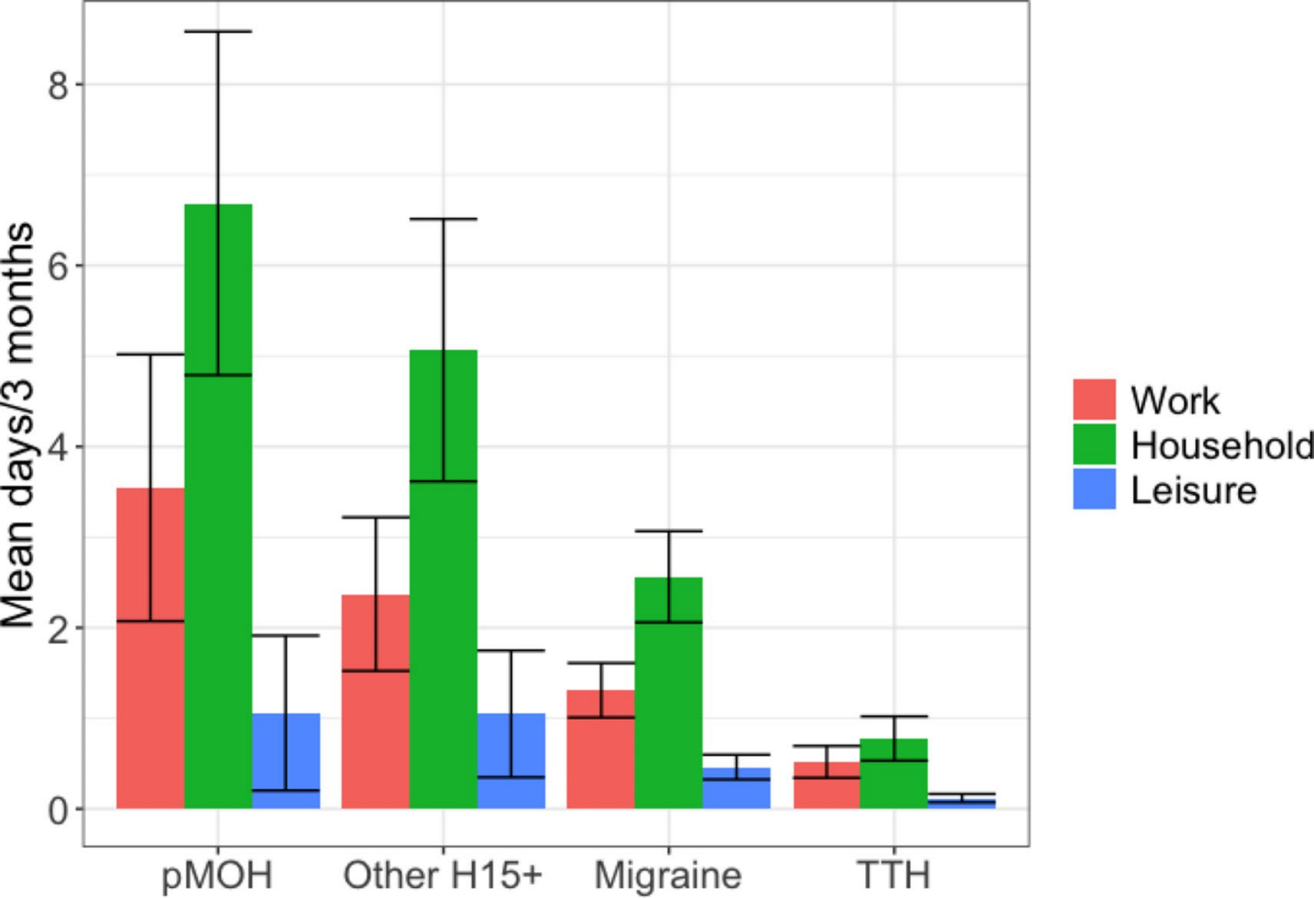
Impaired participation in paid work (red), household work (green) and leisure activity (blue) by headache type. Error bars: 95% confidence intervals; pMOH: probable medication-overuse headache; H15+: headache on ≥ 15 days/month; TTH: tension-type headache

Table 2 and Fig. 1 show headache-attributed impaired participation. Overall, headache of any type was associated with 1.3 lost paid workdays, 2.4 lost household workdays and 0.4 lost leisure days over the preceding 3 months. Household losses were significantly higher among females (2.7 days) than among males (1.7 days; *p* = 0.004). Migraine caused 3–5 times more lost days than TTH, in all three domains and in both genders (Table 2). However, pMOH and other H15 + were associated with even higher losses, pMOH particularly so among females (Table 2). Figure 1 shows clear gradients across headache types, in all domains but especially in work and household days.

**Table 2:** Lost days from paid and household work and leisure activity by headache type and gender

Headache type	Overall	Male	Female	Male vs. female
	Mean±SEM, median			
**HALT1 + 2**				
Any headache	1.3±0.1, 0.0	1.0±0.2, 0.0	1.4±0.1, 0.0	*p* = 0.09
pMOH	3.5±0.7, 0.0	1.6±0.7, 0.0	4.4±1.0, 0.0	*p* = 0.07
Other H15+	2.4±0.4, 0.0	2.8±1.0, 0.0	2.2±0.5, 0.0	*p* = 0.59
Migraine	1.3±0.2, 0.0	1.4±0.4, 0.0	1.3±0.2, 0.0	*p* = 0.62
TTH	0.5±0.1, 0.0	0.5±0.1, 0.0	0.6±0.1, 0.0	*p* = 0.54
	*p* < 0.001			
**HALT3 + 4**				
Any headache	2.4±0.2, 0.0	1.7±0.3, 0.0	2.7±0.2, 0.0	*p* = 0.004
pMOH	6.7±1.0, 0.0	2.6±1.0, 0.0	8.5±1.3, 2.0	*p* = 0.004
Other H15+	5.1±0.7, 0.0	4.9±1.3, 1.0	5.1±0.9, 0.0	*p* = 0.92
Migraine	2.6±0.3, 0.0	2.4±0.6, 0.0	2.6±0.3, 0.0	*p* = 0.77
TTH	0.8±0.1, 0.0	0.8±0.2, 0.0	0.8±0.1, 0.0	*p* = 0.94
	*p* < 0.001			
**HALT5**				
Any headache	0.4±0.1, 0.0	0.4±0.1, 0.0	0.4±0.1, 0.0	*p* = 0.74
pMOH	1.1±0.4, 0.0	0.4±0.2, 0.0	1.4±0.6, 0.0	*p* = 0.28
Other H15+	1.0±0.4, 0.0	2.2±1.3, 0.0	0.7±0.2, 0.0	*p* = 0.06
Migraine	0.5±0.1, 0.0	0.5±0.1, 0.0	0.5±0.1, 0.0	*p* = 0.89
TTH	0.1±0.0, 0.0	0.1±0.0, 0.0	0.1±0.0, 0.0	*p* = 0.88
	*p* < 0.001			

HALT: headache-attributed lost time (questions 1 and 2 relate to paid worktime, questions 3 and 4 to household worktime, question 5 to leisure time); pMOH: probable medication-overuse headache; H15+: headache on ≥ 15 days/month; TTH: tension-type headache

### Burden associated with headache yesterday

Table 3 shows symptom burden and impaired participation associated with HY (*N* = 409). The overall mean duration was 9.1 h, with mean intensity of 1.8 (moderate). Nonetheless, two thirds of those with HY achieved everything as normal (*n* = 167; 40.8%) or more than half (*n* = 100; 24.4%) (Table 3); hence the level of impaired participation in those with HY was 34.7%. No gender-related differences were found in any of these measures.

**Table 3:** Duration and intensity of headache yesterday and its effect on participation

	Overall	Male	Female	Male vs. female
**Duration** (hours)				
mean±SEM, median	9.1±0.5, 4.0	8.9±1.0, 4.0	9.2±0.5, 4.0	*p* = 0.81
**Intensity**				*p* = 0.74
mild (n)	124	33	91	
moderate (n)	231	53	178	
severe (n)	55	13	42	
mean*	1.8	1.8	1.8	
**What done**				*p* = 0.10
everything (n)	167	45	122	
more than half (n)	100	21	79	
less than half (n)	100	18	82	
nothing (n)	42	15	27	

*Equating to 1, 2, 3, and treating as though continuous data

### Quality of life

**Fig. 2:**
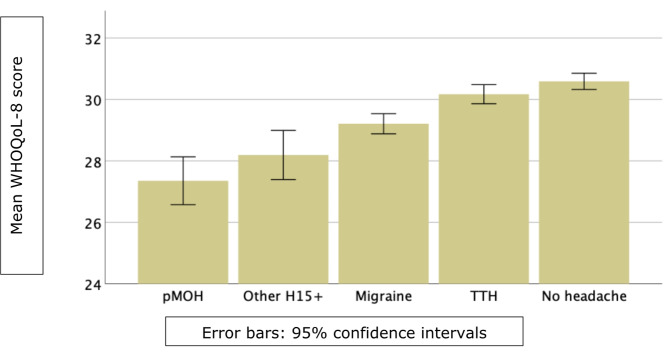
Self-reported quality of life (measured by WHOQoL-8) by headache status pMOH: probable medication-overuse headache; H15+: headache on ≥15 days/month; TTH: tension-type headache

Table 4 and Fig. 2 show self-reported QoL according to headache status. All headache types were associated with diminished QoL, significantly except for TTH. There was a clear gradient downwards (but with, mostly, overlapping CIs) from no headache to TTH to migraine to other H15 + to pMOH.

**Table 4:** Self-reported quality of life (measured by WHOQoL-8) by headache status

Headache status	Quality of life mean±SEM [95% CI], median
No headache	30.6±0.1 [30.3–30.9], 31.0
Probable medication-overuse hedache	27.4±0.4 [26.6–28.1], 28.0
Other headache on ≥ 15 days/month	28.2±0.4 [27.4–29.0], 28.0
Migraine	29.2±0.2 [28.9–29.5], 30.0
Tension-type headache	30.2±0.2 [29.9–30.5], 30.0
	F(4, 4125) = 40.6; *p* < 0.001

### Population-level burden

Table 5 shows pTIS and impaired participation at population level.

**Table 5:** Population-level estimates (age- and gender-adjusted) of proportion of time in ictal state and impaired participation

Headache type	Proportion of time in ictal state (%)		Impaired participation			
	Based on frequency and usual duration (30-day recall)	Based on headache yesterday (1-day recall)	Based on HALT data (90-day recall) (lost days/person/3 months)			Based on headache yesterday (1-day recall) (%)
			Lost productivity		Leisure	
			**Paid work**	**Household work**		
Any headache	6.2	7.4	0.8	1.4	0.3	6.7
Migraine	1.7		0.3	0.6	0.1	
TTH	1.0		0.1	0.2	0.0	
pMOH	2.1		0.2	0.3	0.0	
Other H15+	1.0		0.1	0.2	0.1	

TTH: tension-type headache; pMOH: probable medication-overuse headache; H15+: headache on ≥ 15 days/month

Based on 1-year prevalence, frequency and usual headache duration, and adjusted for age and gender, an estimated 6.2% of all time in the adult general population was spent with headache. Based on 1-day prevalence and duration of HY, this proportion increased to 7.4%. Most time was spent with pMOH (2.1%), followed by migraine (1.7%) and then TTH and other H15+ (both 1.0%).

From HALT data, impaired participation at population level was measured as 0.8 and 1.4 days/person/3 months lost from paid and household work respectively, but only 0.3 lost leisure days. Of all headache types, migraine caused the biggest losses in both paid (0.3 days) and household work (0.6 days), followed by pMOH (0.2 work and 0.3 household lost days/person/3 months). TTH and other H15 + each caused lesser detriments (0.1 work and 0.2 household lost days/person/3 months).

Based on HY data, overall impaired participation in the adult general population attributed to headache was estimated as 6.7%.

### Health-care needs assessment

One third (34.4%; 703/2,041) of the participants had headache likely to benefit from health care: all those with H15+, 370 with migraine and 88 with TTH (Table 6). Adjusting for age and gender, we estimated that 33.2% of the general population aged 18–65 years in Mongolia had need for headache-related health care, according to our definition of “need”: 18.2% for migraine, 4.3% for TTH, 5.7% for pMOH and 5.0% for other H15+.

**Table 6:** Health-Care needs assessment

Criterion fulfilled		Proportion of sample		Estimated proportion of adult population*
		**n**	%	% [95% CI]
1	Headache on ≥ 15 days/month	245	12.0	10.7 [9.4–12.1]
2	Migraine on ≥ 3 days/month	322	15.8	15.0 [13.5–16.6]
3	Migraine and pTIS > 3.3% and moderate-severe intensity	135^1^	6.6	6.3 [5,3-7.5]
4	Migraine and lost work and/or household days/3 months ≥ 3	151^2^	7.4	6.8 [5.8-8.0]
5	TTH and pTIS > 3.3% and moderate-severe intensity	34	1.7	1.6 [1.1–2.3]
6	TTH and lost work and/or household days/3 months ≥ 3	61^3^	3.0	2.8 [2.1–3.6]
One or more of criteria 1–6		703	34.4	33.2 [31.2–35.3]

*Age- and gender-corrected; ^1^of whom 116 also fulfilled criterion 2; ^2^of whom 116 also fulfilled criterion 2, 57 also fulfilled criterion 3 and 51 also fulfilled criteria 2 and 3; ^3^of whom 7 also fulfilled criterion 5; pTIS: proportion of time in ictal state; TTH: tension-type headache

## Discussion

This is the first paper to report headache-attributed burden in Mongolia. It adds to our earlier paper on prevalence [13], using data collected in the same study from the same participants.

In summary, recalling frequency, usual duration and usual intensity over the preceding 30 days, study participants with headache (66.1% of the sample) reported an average of 9.7% of all their time with headache rated mild-to-moderate. Headache yesterday, when reported, was on average rated moderate. Those with migraine reported higher symptom burden than those with TTH, but, at individual level, pMOH and other H15 + far outweighed migraine. Multiplying pTIS for migraine by the ictal state DW from GBD [18] gave a value of 3.0%. Despite the terminology used by GBD, this is an estimate of lost health rather than disability; in other words, the intermittent symptoms of migraine were equivalent to a continuous health diminution of 3.0%. Consequential impaired participation, estimated from recall over the preceding 3 months, was 1.3 days in paid work, 2.4 days in household work and 0.4 days in leisure activity, with a clear gradient between headache types (pMOH > other H15 + > migraine > TTH) in both genders. QoL estimates (despite the non-specificity for headache of WHOQoL-8) reflected symptom burden and impaired participation, with a clear downward gradient from no headache to TTH to migraine to other H15 + to pMOH. These measures show high individual burden from prevalent disease.

A main purpose of this study was to provide population-level estimates to inform health policy. An average pTIS of 9.7% among all headache sufferers diluted to a population-level pTIS of 6.2% when factoring in 1-year prevalence and correcting for age and gender. Over half of this was attributable to pMOH (2.1%) and other H15+ (1.0%), a quarter (1.7%) to migraine. The calculation based on HY for all headache yielded a similar, albeit slightly higher, estimate of 7.4%, indicating robustness of these estimates.

Of particular economic interest are the population-level estimates of impaired participation. On average, each adult person (with or without headache) lost 0.8 workdays and 1.4 household days to headache over a period of 3 months, migraine rather than pMOH being the principal contributor in both cases (more is said about this later). These losses – from work and household chores – can be interpreted as productivity losses, while those from paid work may translate into losses from gross domestic product (GDP). If 3 months are assumed to equate to 65 workdays, an estimated 1.2% of all workdays (0.8/65) are lost to headache. It is worth noting that the individual data on impaired participation were heavily skewed, indicating a severely affected minority for whom targeted intervention might be considered an economic priority.

From HY data we could estimate only total impaired participation across all three domains (paid work, household work and leisure), with limited ability to infer how much of the 6.7% impairment at population level pertained to income-generating work. However, given that most days in a week for most people are workdays, this finding of 6.7% overall impairment suggests that the 1.2% workday loss derived from HALT (dependent on 90-day recall) is an underestimate. Of course, the estimate from HY was based on a much lower N, but it remained acceptable (409) since the original sample was large.

Migraine was associated with the largest detriment in productivity at population level despite that pMOH was associated with higher population-level pTIS (2.1% vs. 1.7%) and higher individual-level burden (in other words, time with pMOH was rated worse than time with migraine). It appears that, during an attack, migraine nonetheless had greater impact on productivity. Associated symptoms (particularly nausea) might have been partly responsible, but a plausible explanation is that people with highly frequent headache develop coping mechanisms, while occasional unpredictable attacks are highly disruptive.

Pursuing the purpose of informing national health policy, our needs assessment found that one third (33.2%) of the adult Mongolian population were likely to benefit from – and were therefore in need of – headache-related health care. This high proportion was mainly driven by the high prevalences of H15+ (10.7%) and migraine on ≥ 3 days/month (15.0%). TTH contributed relatively little (4.3%). We have previously discussed our criteria for defining “need” in this context [22], some of which, arguably, are arbitrary. However, it is uncontroversial that all of those with H15 + should be offered care, and the same appears true for those with migraine on ≥ 3 days/month since this is often considered the threshold for initiating preventative medication. If all other criteria were dropped, an estimated one quarter (25.9%: calculation not shown) of the adult population in Mongolia would still be considered to need headache-related health care.

### Strengths and limitations

As previously noted [13], the strengths of this survey included conduct in accordance with standardized methods [14, 15] in a large (*N* = 2,043) and nationally representative sample with a very high participating proportion (98.3%). Additionally, we estimated headache-attributed burden and impaired participation from data derived from both the preceding day and 1–3 months.

Limitations were those inherent in all cross-sectional retrospective studies. We had a preponderance of females in our sample, but estimates were corrected for gender and age. In addition, there was oversampling of people with high levels of education [13], a factor associated with higher prevalence of migraine but lower prevalence of pMOH [13].

## Conclusions

This first population-based study on headache burden in Mongolia, with the aim of informing health policy, shows that 6.2–7.4% of all time in the adult population is spent with headache. With its very high prevalence and high individual burden, H15 + causes more burden at population-level than migraine and TTH combined. Migraine, however, has greatest negative impact on the nation’s productivity. An estimated one third of the adult population have a headache disorder (mostly H15 + or migraine) likely to benefit from health care. From a purely economic perspective, Mongolia, with limited health resources, would probably be best served by focusing on mitigating migraine-attributed burden.

## Data Availability

The data are held on file at Mongolian National University of Medical Sciences and at Norwegian University of Science and Technology. Once analysis and publications are completed, they will be freely available for non-commercial purposes to any person requesting access in accordance with the policy of the Global Campaign against Headache.

## Abbreviations

ANOVA: Analysis Of Variance
CI: Confidence Interval
DW: Disability Weight
GBD: Global Burden of Disease
GDP: Gross Domestic Product
HARDSHIP: Headache-Attributed Restriction, Disability, Social Handicap and Impaired Participation Questionnaire
ICHD: International Classification of Headache Disorders
HALT: Headache-Attributed Lost Time
HY: Headache Yesterday
MNUMS: Mongolian National University of Medical Sciences
MOH: Medication-Overuse Headache
pMOH: probable MOH
pTIS: proportion of Time In Ictal State
QoL: Quality of Life
SEM: Standard Error of the Mean
TTH: Tension-Type Headache
WHO: World Health Organization
WPR: Western Pacific Region
